# Nest excavators’ learning walks in the Australian desert ant *Melophorus bagoti*

**DOI:** 10.1007/s10071-024-01877-3

**Published:** 2024-05-24

**Authors:** Sudhakar Deeti, Donald James McLean, Ken Cheng

**Affiliations:** School of Natural Sciences, Macquarie University, Sydney, NSW 2109 Australia

**Keywords:** Nest excavation, Navigation, Red honey ant, Exploratory walks

## Abstract

The Australian red honey ant, *Melophorus bagoti*, stands out as the most thermophilic ant in Australia, engaging in all outdoor activities during the hottest periods of the day during summer months. This species of desert ants often navigates by means of path integration and learning landmark cues around the nest. In our study, we observed the outdoor activities of *M. bagoti* workers engaged in nest excavation, the maintenance of the nest structure, primarily by taking excess sand out of the nest. Before undertaking nest excavation, the ants conducted a single exploratory walk. Following their initial learning expedition, these ants then engaged in nest excavation activities. Consistent with previous findings on pre-foraging learning walks, after just one learning walk, the desert ants in our study demonstrated the ability to return home from locations 2 m away from the nest, although not from locations 4 m away. These findings indicate that even for activities like dumping excavated sand within a range of 5–10 cm outside the nest, these ants learn and utilize the visual landmark panorama around the nest.

**Supplementary Information:**

The online version contains supplementary material available at 10.1007/s10071-024-01877-3.

## Introduction

The ecological success of social insects, notably ants, is frequently attributed to the division of labour (Hölldobler and Wilson 1990). Individuals in a colony specialize in specific tasks, such as caring for the brood, foraging, constructing nests, or defending the colony (Wilson 1987). Each task is carried out by a distinct subset of the worker population. Specialization is found even when all the workers look similar. Colonies of the Australian desert red honey ant, *Melophorus bagoti*, exhibit monomorphic workers, with a similar physical appearance across workers (Deeti and Cheng 2021a). Despite this uniformity, there exists specialization in tasks and roles among these monomorphic workers. Our personal observations suggest that red honey ant workers engage in three distinct outdoor activities. Firstly, nest excavation is a continuous activity that persists from the colony’s post-hibernation phase until it resumes hibernation over the winter. This activity involves the construction and maintenance of the nest structure, including taking excess sand out of the nest. Secondly, foraging activity is performed by a subset of workers. These individuals scavenge for dead arthropods and seeds in the desert terrain, transporting their findings back to the nest (Muser et al. 2005; Schultheiss and Nooten 2013). Lastly, dumping activity involves certain workers disposing of food waste outside the nest before returning to the colony (Deeti et al. 2023a). Aside from foraging behaviour, however, the other two activities, namely nest excavation and dumping, remain understudied in this desert ant. A separate study or ours examines the paths of dumpers, both experienced and naive (Deeti et al. 2024a). Our focus here is on comprehending the navigational understanding and learning of excavation workers.

Desert ants have been extensively studied for their remarkable navigational abilities, particularly within the genera *Cataglyphis*, *Ocymyrmex*, and *Melophorus* (Wehner 2020). These ants, known for their visual navigation skills during foraging periods outside the nest, possess a diverse navigational toolkit. This toolkit encompasses path integration, enabling ants to continuously track the direction and distance to their starting position (Collett and Collett 2000; Wehner and Srinivasan 2003). This ability enables an animal to turn and orient itself towards a starting point, such as its nest, without relying on terrestrial visual cues or knowledge of the nest’s surroundings. Ants, however, also learn the surrounding terrestrial visual cues for navigation. Perceived views are matched to learned and remembered views to navigate home (Cheng 2012; Knaden and Graham 2016; Wehner 2020). The toolkit also includes systematic search strategies (Schultheiss et al. 2015; Wehner and Srinivasan 1981) and backtracking capabilities (Wystrach et al. 2013). The integration of these sophisticated navigational tools highlights the flexibility of desert ants in navigating their environments (Hoinville and Wehner 2018; Wehner et al. 2016). Some parts of the toolkit require some learning to set up; this is especially so for view-based navigation by foragers. Characteristic ‘learning walks’ performed before heading off on foraging journeys are thought to facilitate the learning of visual cues (Knaden and Graham 2016; Wehner 2003; Wehner et al. 2004).

Venturing beyond the nest’s entrance exposes insects such as ants to a higher risk of predation and getting lost. To mitigate the risk of getting lost, foragers engage in 3–7 learning walks around their nest before heading off on any extended journey (Deeti and Cheng 2021b; Fleischmann et al. 2016; Jayatilaka et al. 2018; Freas et al. 2019; Zeil and Fleischmann 2019). These pre-foraging walks consist of loops of increasing size, covering a larger area with each successive walk. This process plays a vital role in enabling ants to familiarize themselves with the visual landscape surrounding their nest. View-based models of ant navigation propose the existence of a catchment area around the nest, where views acquired during learning walks guide foragers back to the nest (Zeil 2012; Zeil et al. 2003, 2014). Studies on *Cataglyphis noda* and *Melophorus bagoti* reveal their ability to generalize views to non-visited locations up to 10 m away after learning the nest panorama in a limited area around their nest entrance (Deeti et al. 2020; Fleischmann et al. 2018; Wystrach et al. 2012). Previous observations of *M. bagoti* also revealed that, after a single initial learning walk within a range of ~ 20 cm, they could generalize their views to find their way back to the nest from a distance of 2 m but not 4 m (Deeti and Cheng 2021b). Earlier observations on *M. bagoti* also highlighted the importance of learning walks during the first two days of outdoor life for nest finding (Muser et al. 2005). Despite these insights, additional research is crucial to fully comprehend the developmental aspects of learning walks for all worker castes in desert ants. In *M. bagoti*, it remains uncertain whether those desert ants engaged in daily nest excavation activities require learning walks for this task and to what extent they generalize views during this process. We did not expect excavating ants to do any learning walks because our companion study conducted earlier on dumpers (Deeti et al. 2024a preprint), which travel greater distances than excavators, showed that dumpers at one nest did not do any learning walks, and because the distance travelled by excavators is small, not more than 15 cm. To foreshadow, however—a point that will be obvious in the next paragraph and the methods—we were wrong.

In our current study, we examined the learning walks of nest-excavating red honey ants, specifically focusing on their pre- and post-excavation activities in their natural habitat. We documented the structure and spatial distribution of learning walks and excavation activities conducted by red honey ants at the nest site. Moreover, we carried out displacement experiments following the ants’ initial excavation walk to evaluate their ability to effectively use the terrestrial panorama from a greater distance to the nest.

## Methods

During the Australian summer months from November 2023 to December 2023, we conducted a study on *Melophorus bagoti* Lubbock 1883 desert ants originating from a single nest situated in the vicinity of the Centre for Appropriate Technology, located 10 km south of Alice Springs, NT, Australia (23°45′28.12″S, 133°52′59.77″E). The prevalent vegetation in this semi-arid desert habitat includes buffel grass (*Pennisetum cenchroides*), a mosaic of *Acacia* bushes, and *Eucalyptus* trees. No specific ethical regulations regarding the study of ants are found in Australia, and all experimental procedures employed were entirely non-invasive.

### Animals

The red honey ant *Melophorus bagoti* is the most thermophilic ant on the Australian continent (Christian and Morton 1992), engages in three outdoor activities during hot summer days, and forages by primarily scavenging deceased arthropods while collecting sugary plant exudates and seeds (Muser et al. 2005; Schultheiss and Nooten 2013). Besides foraging, some workers dedicate themselves to excavating the nest throughout the day while others dump waste materials from the nest. Foraging ants operate individually for short durations, covering distances up to 50 m from the nest, relying on path integration and terrestrial visual landmarks without utilizing any chemical trails (Cheng et al. 2009). In contrast, nest workers involved in excavation activities remove sand from the nest, depositing it within a 15-cm radius outside (Deeti and Cheng 2023a). Some of the ants bigger in size also guard the nest entrance and occasionally move around the nest.

Our investigation required the examination of naive excavating ants. Workers start foraging after completing tasks within the nest. Once outside, they lasted on average 4.9 days before they disappeared and were presumed to have died (Muser et al. 2005). To identify the naive ants, all workers emerging from the colony were uniformly painted with the same colour over a 6-day period to ensure that all experienced ants were marked (Deeti and Cheng 2021b). From the 7th day onward, any unpainted ants emerging from the nest were considered as newcomers with no prior experience of terrestrial landmarks around their nest. These ants were captured at the nest entrance immediately upon appearance and marked with an individually distinct colour code on the abdomen or thorax (Tamiya™) (Deeti and Cheng 2021a). We painted 40 naive ants, and following the marking process, naive ants were released back into the nest entrance. To prevent them from re-emerging, the nest entrance was securely sealed with a lid until the ant went into the nest (about 20 s) (Deeti and Cheng 2021b). Usually nest activity started at ~ 9:00 in the morning and ended at ~ 17:00 in the evening. We observed the nest during ‘working hours’ but otherwise left the nest alone.

### Experimental procedure

The vegetation surrounding the chosen nest was cleared on the initial day of the study. To enhance the visibility of the red ants against the sandy red soil background, fine white sand was spread within the recording area. This ensured a clear contrast between the ants and the background, facilitating easy distinction in the recorded footage. Out of the total 40 painted naive ants, the study focused on the 20 ants that worked on nest excavation after their initial learning walk, and displacement experiments were conducted on these same 20 marked individuals known to be naive excavating ants. As it turned out, upon their first reappearance, these newly marked (painted) naive ants that went on to excavate always exhibited a small loop around the nest. These naive walks were recorded with a video camera (Sony Handy camera (FDR-AX700), recording at a frame rate of 25 frames per second. A tripod was set at a height of 1.2 m from the ground, with the camera looking straight down at the ground, and the recording area measured 1 m by 1 m centred at the nest, and the camera boasted a resolution of 3860 by 2160 pixels. After this initial walk, in their next appearance, when, as it always turned out, they deposited sand outside and returned to the nest, they were captured before entering and displaced to four test locations at 2–4 m in distance, positioned to the North and East of the nest. These directions were chosen because they possessed distinctive panoramas compared to the other cardinal directions. Each ant underwent testing at each of these four locations (2mN, 2mE, 4mN, 4mE) in a random order. At the displacement site, ant trajectories were recorded. Once the tests were completed, we released the test ant back into the nest. In subsequent appearances, they were observed carrying excavated sand in their mandibles and typically tossing it outside the nest within a range of 5–10 cm. We also video-recorded the excavation activity of these ants at the nest after their displacement tests.

Test ants were manually captured near their nest using a wide-mouth 50-ml glass container, transported in darkness, and released at the centre of the recording area. Each ant was released one at a time at the displacement site. After leaving the recording area of the first test, the ants were captured in a test container and then released at the next site. Once an ant finished all four displacement tests, it was released at the nest vicinity to return home, which all of them did.

### Tracking

We used the animal tracking program DLTdv8 (version 8.2.9) in MATLAB (2022B) to extract frame-by-frame coordinates of the head and thorax — specifically the tip of the head and the middle of the thorax (see Fig. S1) — for each ant in every video obtained during our displacement test recording as well as the recordings of their learning and excavation walks. These extracted frame-by-frame coordinates served as the basis for all subsequent analyses of the workers’ movements and behaviour.

### Data analysis

In this study, we analysed the ants’ orientation at the displacement site and path characteristics while performing the excavation. On displacement tests, the final heading direction of ants was determined by assessing the thorax–head direction vector as they exited the recording area, that is, on the last frame in which they were visible. This vector represents the orientation of each ant at that specific moment. To analyse and visualize this data, the final heading mean vector angles were divided into 24 equal wedges, each spanning a 15-degree angle. These wedges are employed in circular plotting to illustrate how directly oriented towards the nest the ants’ trajectories were at the displacement site. For analysis, we used the circular statistics package in R (version 4.2.1; R Core Team, 2020) on the exact exit headings of the ants (and not the wedge-sector headings). We analysed the maximum displacement from the nest, the duration and the area covered by the ants’ trajectory. The nest location was chosen as the origin (0, 0). These measures helped us understand how extensively the ants explored their surroundings during the learning walks and in the case of excavation walks, discern any notable differences compared to their learning walks. Maximum displacement was the thorax position at the maximum distance from the nest. We recorded the duration of each learning and excavation walk from the moment the ant left the nest until just before it entered the nest. For the area covered during these walks, we calculated the enclosed area of the path joining the thorax positions of the ants, using the convex-hull method to encompass all the points visited by the ant. This area measurement provided a measure of the spatial extent of their exploration.

To understand how quickly and directly the ants moved on each learning and excavation walk, we calculated speed of the workers along with several characteristics of the trajectory. These path characteristics were based on the positions of the thorax across frames. Speed refers to the magnitude of an ant’s velocity and was calculated as the average over the entire trajectory for each ant, excluding the stopping durations. We measured the speed at which ants changed their orientation direction by observing how quickly their head direction changed over time. As ants walk, their head shifts left and right. To determine orientation direction, we drew a line from the thorax coordinates to the head coordinates. Orientation angular velocity was calculated by dividing the change in orientation angle by the change in time. This helped us understand how fast ants adjusted their viewing direction.

To understand how direct an ant’s path was, we used three indices of straightness: *path straightness, sinuosity*, and ${E}_{max}^{a}$, each of which relates to the directness of navigation towards a destination. *Straightness* is computed as the ratio of the straight-line distance between the release point at the displacement site and the final point in the frame before the ant moved out of the recording area to the overall length of the path. (Batschelet 1981; Deeti et al. 2023b, 2024b, c; Islam et al. 2021, 2023). *Straightness* ranges from 0 to 1, with larger values indicating straighter paths, while smaller values indicate more curved or convoluted paths. *Sinuosity* is an estimate of the tortuosity in a path, calculated as $S=2{\left[p\left(\frac{1+c}{1-c}+{b}^{2}\right)\right]}^{-0.5}$, where *p* is the mean step length, *c* is the mean cosine of turning angles and $b$ is the coefficient of variation of the step length. A trajectory *step* is the movement between the positions of the animal (thorax positions) recorded at consecutive video frames. Accordingly, step lengths are the Euclidean distances between consecutive points along a path, and turning angle refers to the change in direction between two consecutive steps. Sinuosity varies between 0 (straight) and 1 (extremely curved) (Benhamou 2004). The maximum expected displacement of a path, ${E}_{max}^{a}=\frac{{\beta }}{1-{\beta }}$, where ${\upbeta }$ is the mean cosine of turning angles, is a dimensionless value expressed as a function of number of steps, and is consistent with the intuitive meaning of straightness (Cheung et al. 2007). Larger maximum expected displacement values indicate straighter paths, hence greater displacement, while a smaller value suggests more localized or constrained movement. Paths were characterized and visualized in R (version 4.2.1; R Core Team, 2020) using the packages trajr (McLean and Skowron Volponi 2018) and Durga (Khan and McLean 2023). Statistical analyses were conducted using R (version 4.3.1). During learning walks, ants frequently displayed a series of stereotypical successive fixations in different head directions by rotating on the spot at one location, known as a “scanning bout” and we extracted the number of scanning bouts from each learning walk (Deeti et al. 2023c; Lionetti et al. 2023).

We also compared the learning walks of excavators and the first learning walks of foragers from the same nest. Given that we video-recorded the first walks of all naive ants, we had these future foragers’ first learning walks on video. Firstly, for the area covered during learning walks, we calculated the enclosed area of the path joining the thorax positions of the ants, using the convex-hull method to encompass all the points visited by the ant. Secondly, we analysed the maximum displacement from the nest during each learning walk. The nest location was chosen as the origin (0, 0). These measures helped us understand how extensively the ants explored their surroundings during the learning walks. Maximum displacement was the thorax position at the maximum distance from the nest. We also recorded the duration of each learning from the moment the ant left the nest until it returned to nest or until it exited the recording area.

We obtained panoramic images using Richo Theta cameras at each test location, including one directly on top of the nest. These panoramas were utilized to compute the rotational image difference function (rotIDF) between the nest panorama and each test-location panorama. The pixel differences were calculated for each 1° shift in pixels, following methods outlined in previous studies (Islam et al. 2022). We calculated the depth of a rotIDF as the mean discrepancy minus the minimum discrepancy.

### Statistical analysis

We used generalized linear mixed models to investigate whether the displacement location affected the route navigation of the excavators on tests. Path characteristics were compared across the four different displacement conditions. In the model, displacement conditions were treated as an independent variable (X) that predicts the dependent variables (Y) and Ant ID was treated as a random factor. We used 4 individual models to compare the effect of the displacement location on *path straightness*, *sinuosity*, ${E}_{max}^{a}$, and speed. Similarly, 4 other models were used to compare characteristics of learning walks of excavators and excavation activity, in speed, convex-hull area, maximum displacement, and duration. We conducted a Welch’s ANOVA (one-way) in cases of variables with significant heterogeneity of variance. The GLM was formulated using the lme4 package (version 1.1–27) and fitted using the glmer function (Bates et al. 2015). Since *path straightness* and *sinuosity* are bounded measures (0–1), we used the binomial family, whereas all of our other response variables are only bounded by zero, and so for them we applied the Gaussian family of models. For paths on tests, we employed the Tukey post hoc test (emmeans package), to perform pair-wise post hoc comparisons for each of our models. Since we tested multiple dependent variables computed from the same data sets of trajectories on tests or on walks at the nest, we adopted *p* = 0.01 as an alpha level to lower type 1 errors. We report all significant terms and the significance values for all key pair-wise comparisons. We visualized the data using summary characteristics such as median (Box plots), density and mean confidence intervals (Deeti et al., 2024c).

To compare the learning walks with excavation walks, we examined the first three excavation walks together with the learning walks (one for each ant), so that this factor, the independent variable, contained four levels. The excavation walks all looked similar when we observed them, so we limited the analysis to a small number that every ant in the study carried out. In the generalized linear-model ANOVA, we applied a priori Helmert contrasts. The learning walk was compared against all three excavation walks in the first contrast. The second contrast compared excavation walk 1 against excavation walks 2 and 3 together, and the third contrast compared excavation walks 2 vs. 3. These contrasts are independent, making up the 3 df in the numerator of the ANOVA. Our hypothesis before data analysis was that only the first Helmert contrast would reach significance. For the statistical comparison of path characteristics of excavators and foragers on their first learning walk, we used a t-test. This test compares the means of categorical variables, and we set the significance level (P-value) to 0.01.

To assess the uniform distribution of headings for each test condition (*P* > 0.05), we performed a Rayleigh’s test. In case both distributions turned out non-uniform, we planned to compare the mean direction of the two groups using the Watson–Williams test (alpha = 0.05). If the hypothesis of a uniform distribution cannot be rejected for one or both groups, it makes no sense to run this test. Additionally, we examined if final heading orientations significantly clustered around the nest direction at 0 degrees by checking whether 0 degrees fell within the 95% confidence interval (CI) of orientations (Watson tests). V-tests were conducted, with alpha set at *P* = 0.05, to determine if the mean headings were notably clustered around a specified target direction. Single-sample log likelihood ratio tests were also conducted to investigate whether the heading distributions of the ants were uniform in each test condition.

## Results

**Fig. 1:**
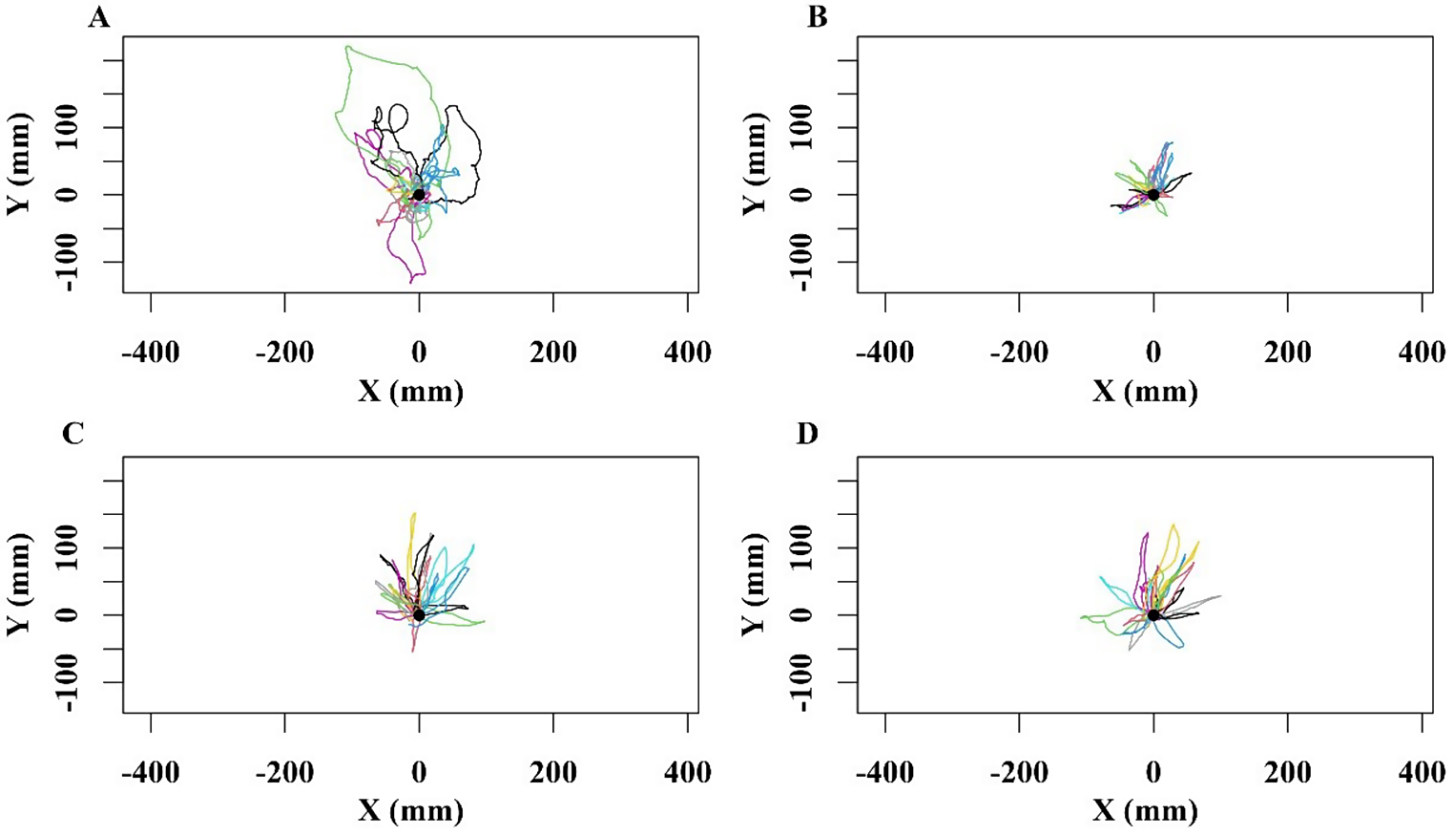
Naive learning walk and nest-excavating ants’ paths. The line graphs show the naïve learning walks of excavators (**A**), each ant represented by a different colour, and same ants’ first (**B**), second (**C**) and third (**D**) round-trips during the sand-excavation. The nest is located at (0, 0)

After emerging from winter hibernation, in the initial days of activity in the new summer season, we observed the naive walks of nest excavators and their subsequent outbound excavating trips. All the ants in this study engaged in a distinctive pattern. They first conducted a single learning walk in the vicinity of the nest. On the same day, following the learning walk, they then actively participated in nest excavation or digging activities. During the learning walk near the nest, the ants walked in a loop in close proximity to the nest and returned to the nest (Fig. 1A). This looping trajectory resembled the pattern observed during foragers’ first, naive learning walks (Deeti and Cheng 2021b). Subsequently, they excavated sand from inside the nest, dumping it outside by tossing the grain, much like the dumping behaviour observed in an earlier study (Deeti and Cheng 2023a). On these excavation trips, we observed the ants to come straight out, drop the sand, and then turn around and head straight back without any ado; except for tossing the sand, they did not stop, and they did not scan on any of these trips.

**Fig. 2:**
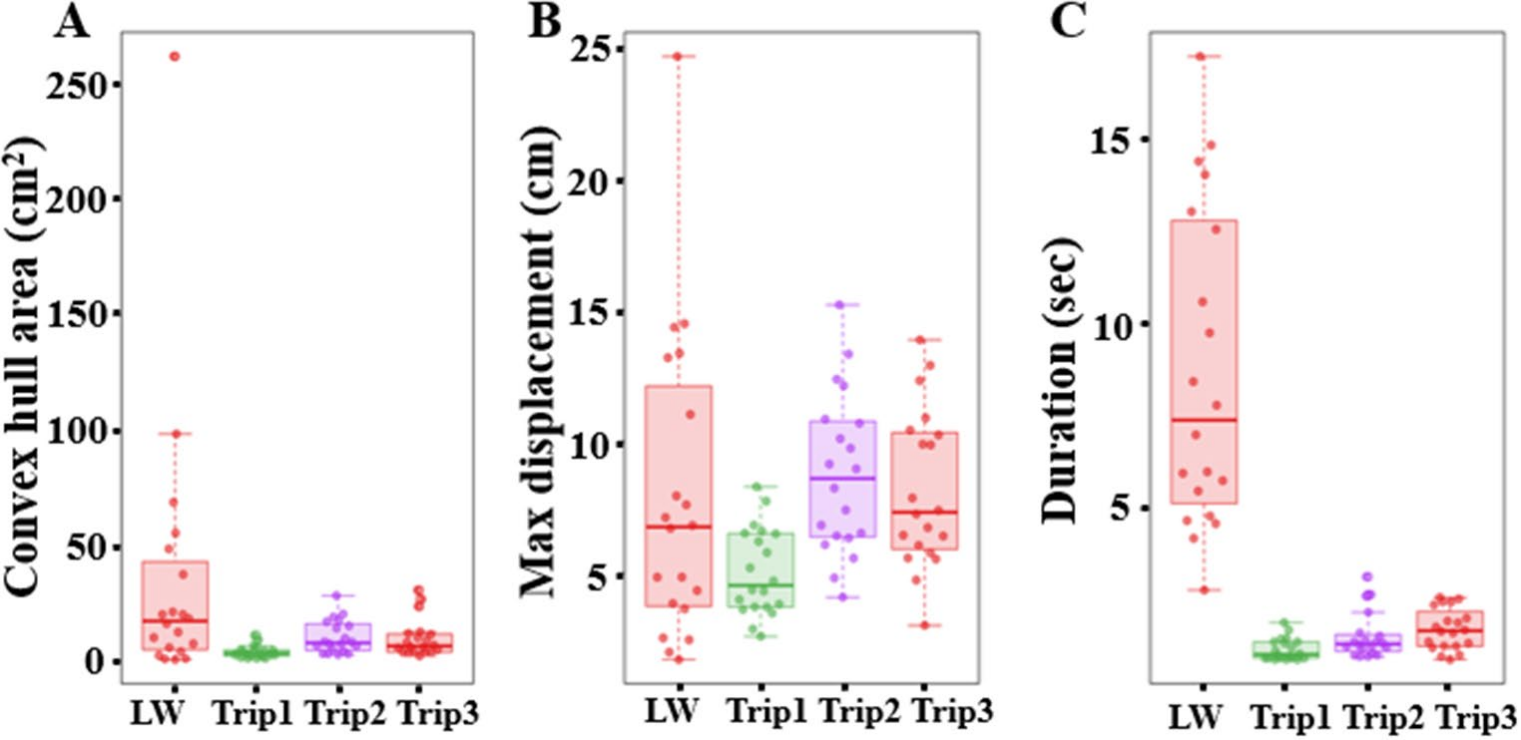
Characteristics of naive learning walks and three consecutive excavation trips of ants at the nest. (**A**) Convex hull area, (**B**) maximum displacement from the nest, and (**C**) duration of the walk. The boxes indicate the median and quartiles, while the whiskers show extreme values excluding outliers. Each point represents a single trajectory. LW on x axis denotes the excavator learning walk

We conducted an analysis of various path characteristics associated with the learning and three consecutive sand excavation walks. Ant paths covered a significantly smaller area during the excavation trips than the learning trip (Fig. 2A). The generalized linear-model ANOVA revealed a significant difference in area between conditions with a priori Helmert contrast-1 (*F*_(1,76)_ = 4.35, *p* = 0.0006), between the learning walk vs. all three excavation walks, but not with contrast-2 (the excavation trip 1 vs. 2 and 3) (*F*_(1,76)_ = 1.4, *p* = 0.14) or contrast-3 (excavation trip 2 vs. 3) (*F*_(1,76)_ = 0.06, *p* = 0.804). In maximum displacement from the nest, the learning walks showed numerically larger values than did the excavation walks (Fig. 2B). A Welch’s one-way ANOVA on the maximum displacement, however, revealed no significant difference between the conditions (*F*_(3,76)_ = 1.04, *p* = 0.06). While there was not a statistically significant difference in maximum displacement between the learning walks and excavation activity, ants spent a longer time outside during the learning walks compared to the excavation activity (Fig. 2C). The generalized linear-model ANOVA found significant differences between the conditions in contrast-1 and contrast-2 (the learning walk vs. all three excavation walks: *F*_(1,76)_ = 4.45, *p* = 0.001; the excavation trip 1 vs. 2 and 3: *F*_(1,76)_ = 6.01, *p* = 0.004) but not in contrast-3, (excavation trip 2 vs. 3: *F*_(1,76)_ = 0.06, *p* = 0.84). During the learning walks, ants stayed for a longer duration outside the nest and covered a larger area than during the excavation trips. A Welch’s one-way ANOVA on convex-hull area covered by walks revealed a significant difference between the conditions (F_(1, 35)_ = 17.2, *p* = 0.00001). The generalized linear-model ANOVA found significant differences between the conditions in contrast-1 (*F*_(1,76)_ = 33.18, *p* = 0.00001), but not in contrast-2 (the excavation trip 1 vs. 2 and 3: *F*_(1,76)_ = 1.96, *p* = 0.056) or in contrast-3 (excavation trip 2 vs. 3: *F*_(1,76)_ = 1.06, *p* = 0.3). Thus, the ants moved similarly far from the nest on learning walks and excavation trips, but learning walks were longer in duration and covered more area.

**Fig. 3:**
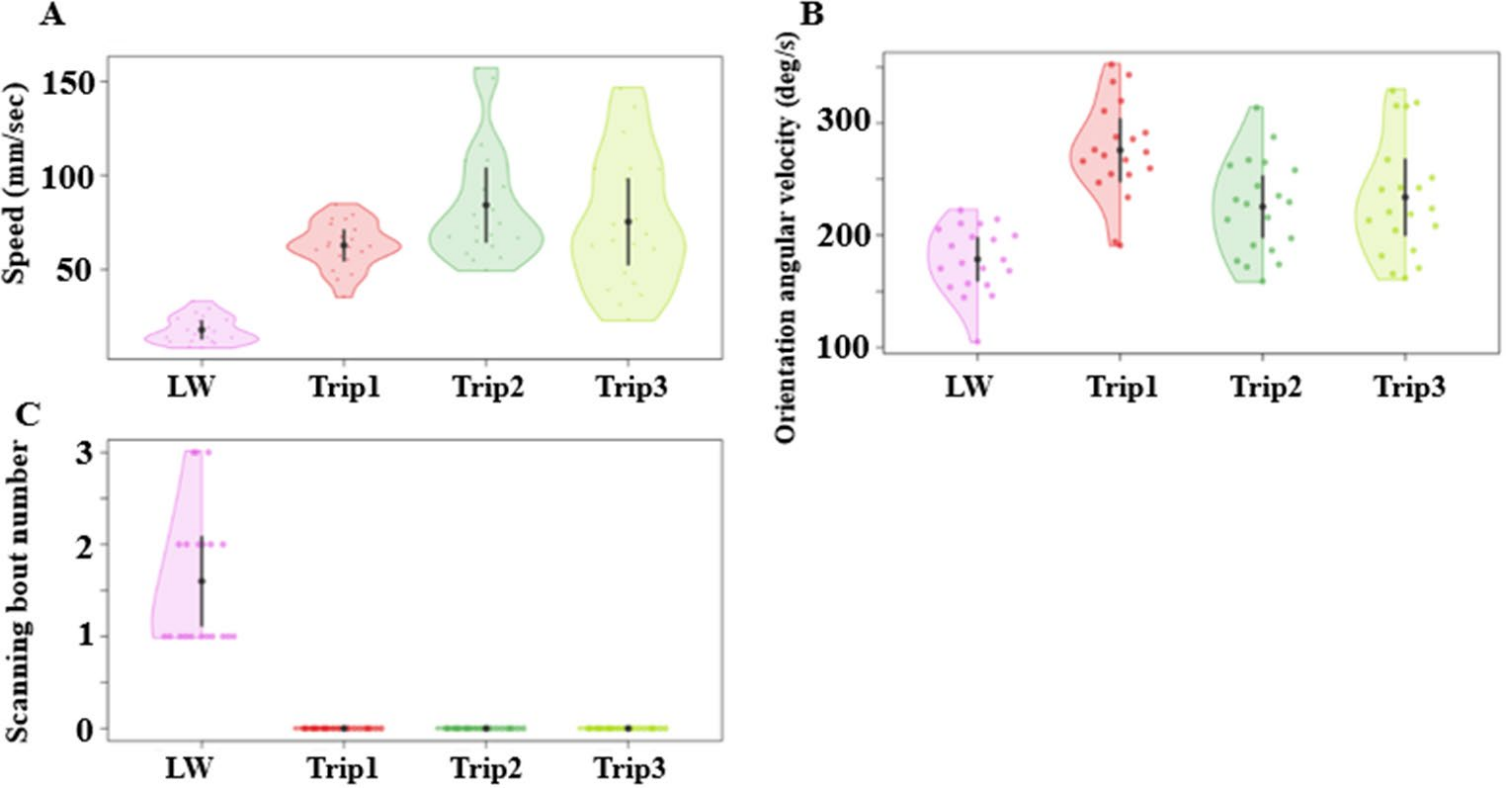
Comparison of speed, orientation angular velocity and scanning bouts of excavators during the learning walk and three consecutive excavation trips. The violin plots show the mean speed of ants across their entire trajectory (**A**). The half violin plots show the distribution of bootstrapped differences of mean orientation angular velocity of ants during the learning walk and consecutive excavation trips (**B**). Number of scanning bouts of naive ants during the learning walk and next three consecutive excavation trips (**C**). In all graphs, the solid dot shows the mean, while the vertical bar shows 95% confidence interval of the mean

We found a notable difference in mean speed between the excavation activity and the naive learning walk. The mean speed during the learning walk was significantly slower than the speed during the excavation activity (Fig. 3A). The a priori Helmert contrast-1 revealed a significant difference (*F*_(1, 76)_ = 30.33, *p* = 0.0005). Other Helmert contrasts, however, revealed no significant differences between the conditions in contrast-2 (*F*_(1,76)_ = 3.7, *p* = 0.012) or contrast-3 (*F*_(1,76)_ = 1.01, *p* = 0.32). In the angular velocity of orientation-direction change, the ants showed a numerically lower angular velocity during the learning walks than during the excavation trips (Fig. 3B). This difference resulted in a statistically significant difference between conditions in a priori Helmert contrast-1 (*F*_(1, 76)_ = 4.52, *p* = 0.005), with no significant differences in contrast-2 (*F*_(1, 76)_ = 0.43, *p* = 0.64) or contrast-3 (*F*_(1, 76)_ = 0.33, *p* = 0.56). During the learning walks, ants showed scanning behaviour. Naive learners performed a minimum of one scanning bout and a maximum of 3 bouts during the learning walks, but no ant scanned during any of the excavation trips (Fig. 3C), making inferential statistics on this variable both inappropriate and moot.

**Fig. 4:**
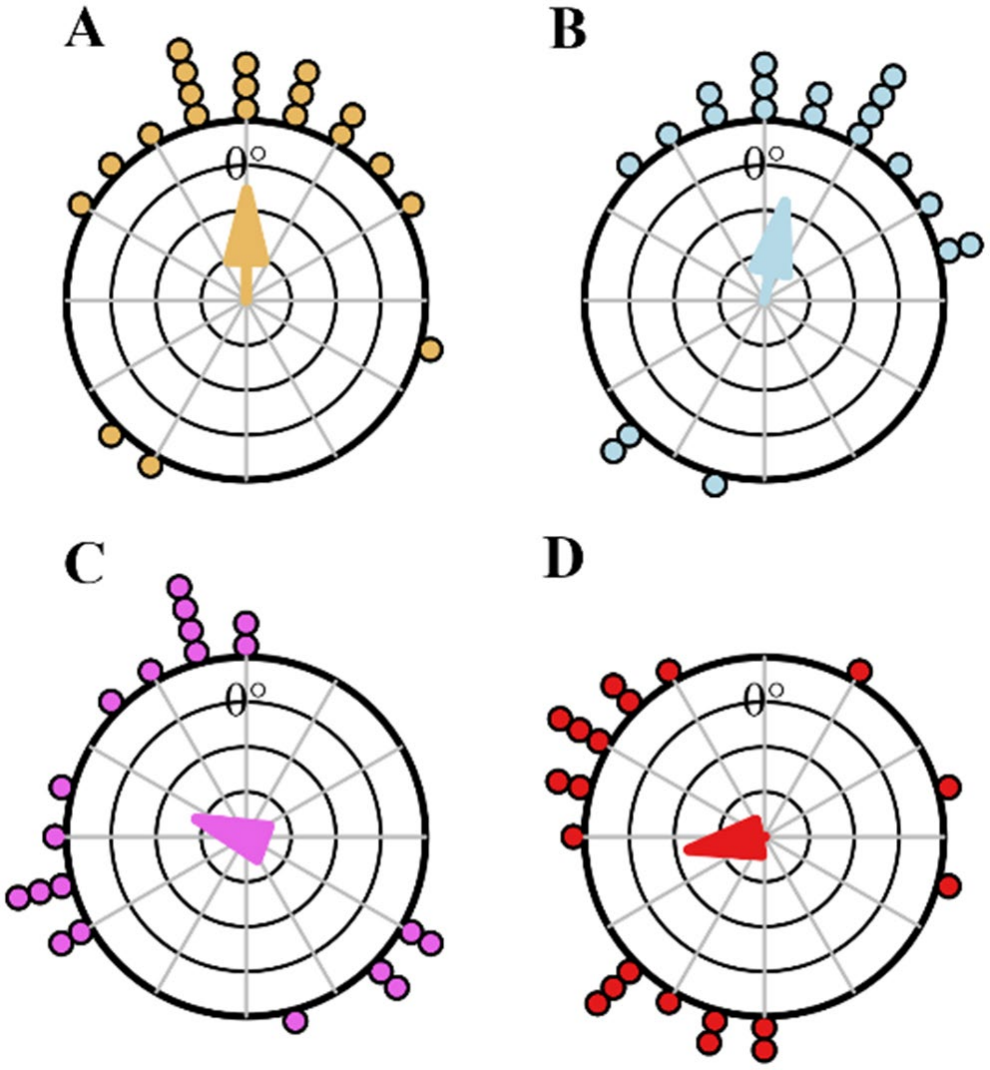
Circular histograms of initial headings of foragers during the displacement test on North 2 m (**A**), East 2 m (**B**), North 4 m (**C**) and East 4 m (**D**) tests. In the histograms, the nest direction is set at 0°. The arrows denote the length and direction of the mean vector

In order to understand the navigational knowledge of these excavators, after their first excavation trip, each ant was captured just before it entered the nest and displaced to four different locations to the North and East of their nest entrance. The ants’ final headings at each location showed that the majority of the ants in the 2-m displacements were oriented towards the nest direction of 0 deg. In contrast, ants that were displaced 4 m from the nest were not oriented towards the nest direction from the release point (Fig. 4). By the Rayleigh test, the foragers’ initial orientations were non-uniformly distributed in 2-m displacement tests whereas in 4-m tests they were scattered and uniformly distributed (Table 1; Fig. 4). In addition, ants in 2-m displacement conditions showed significant V-test results in the nest direction, and the means of their 95% confidence interval of initial heading values include the nest direction 0 deg (Watson test, *p* > 0.05). The log likelihood ratio test for the 2-m tests failed to reject the hypothesis that the distribution was clustered in the home direction (*p* ≥ 0 0.05, k ≥ 0, χ2 ≤ 1). For the 4-m test, however, the log likelihood ratio rejected the hypothesis that the mean value of distribution was equal to the predicted value (home direction) (*p* = 0.003, k = 0.56 and χ2 = 4.6), meaning that the headings were oriented in a different direction from the nest direction.

**Table 1:** Statistical results for initial heading directions in 2 m North, 2 m East, 4 m North and 4 m East displacement tests

	Mean vector	95% confidence interval		Rayleigh test		V test detection 0^*0*^	
Test	µ	Minus	Plus	Z	P	Z	P
2 m North	0.34°	335.67°	5.01°	7.96	< 0.001	3.91	< 0.005
2 m East	11.95°	344.05°	39.85°	6.60	< 0.001	3.55	< 0.002
4 m North	289.77°	232.45°	317°	1.83	0.16	0.64	0.26
4 m East	260.11°	221.71°	298.51°	3.84	0.01	-0.47	0.68

**Fig. 5:**
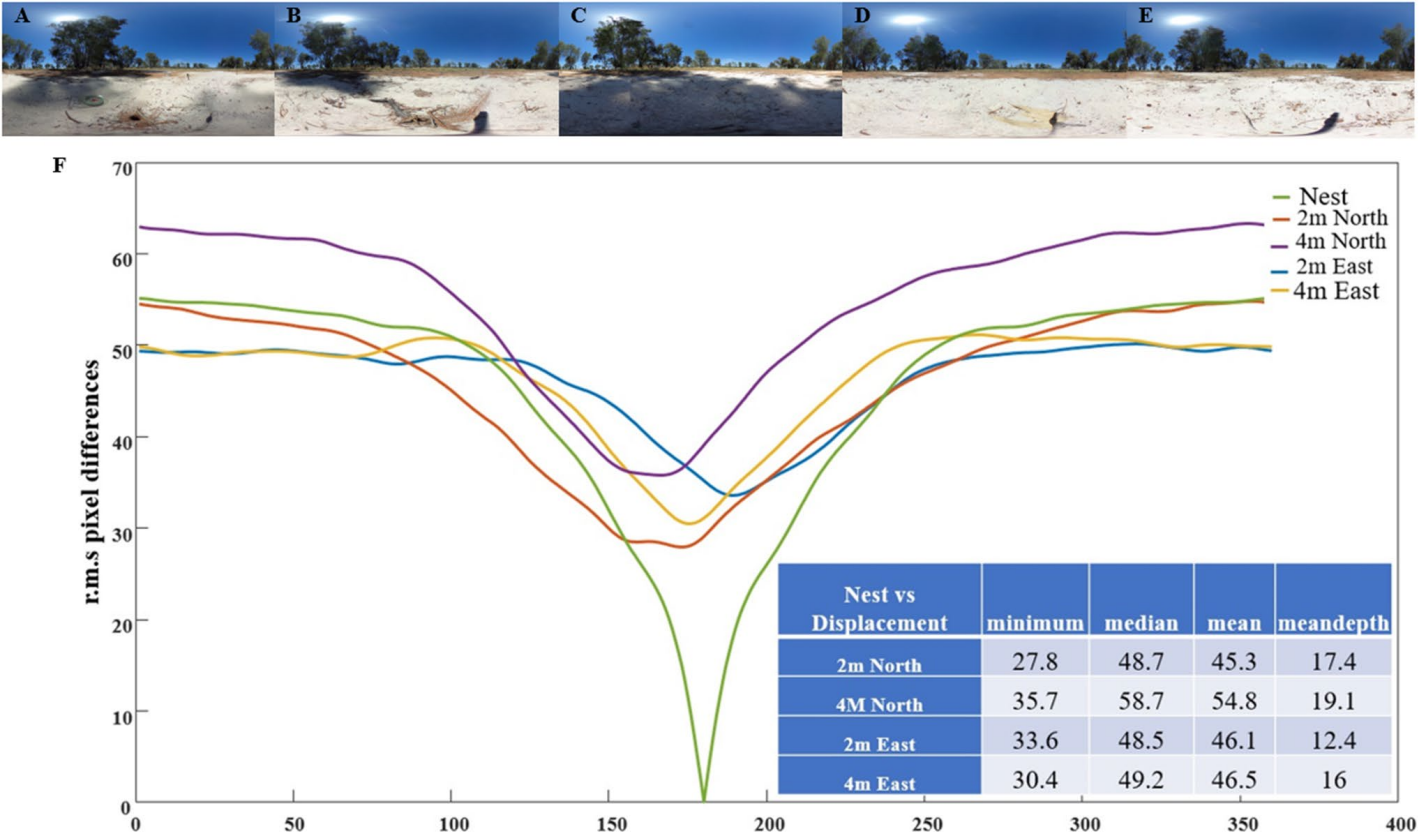
(**A** to **E**) Panoramic views at various tested locations, all aligned towards the nest direction: at (**A**) the Nest, (**B**) 2 m North, (**C**) 4 m North, (**D**) 2 m East, and (**E**) 4 m East. **F**). The rotational image difference function for each location when compared with the nest panorama facing the direction of the nest. Table in the inset shows the minimum, median, mean and mean depth at each test location when compared with the nest panorama facing the direction of the nest

We utilized rotIDF to assess the visual similarity of each displacement location to the nest panorama. Comparisons with the nest panorama revealed detectable minima for all displacement locations, with specific values 2 m North location = 27.88, 4 m North location = 35.73, 2 m East, location = 33. 61, 4 m East = 30.49 (Fig. 4). Interestingly, the depth of these minima showed only slight variation across displacement locations: 2 m North location = 17.49, 4 m North location = 19.14, 2 m East, location = 12.48, 4 m East = 16.04 (Fig. 5). This suggests that all test locations had a ‘best’ direction pointing roughly towards the nest.

**Fig. 6:**
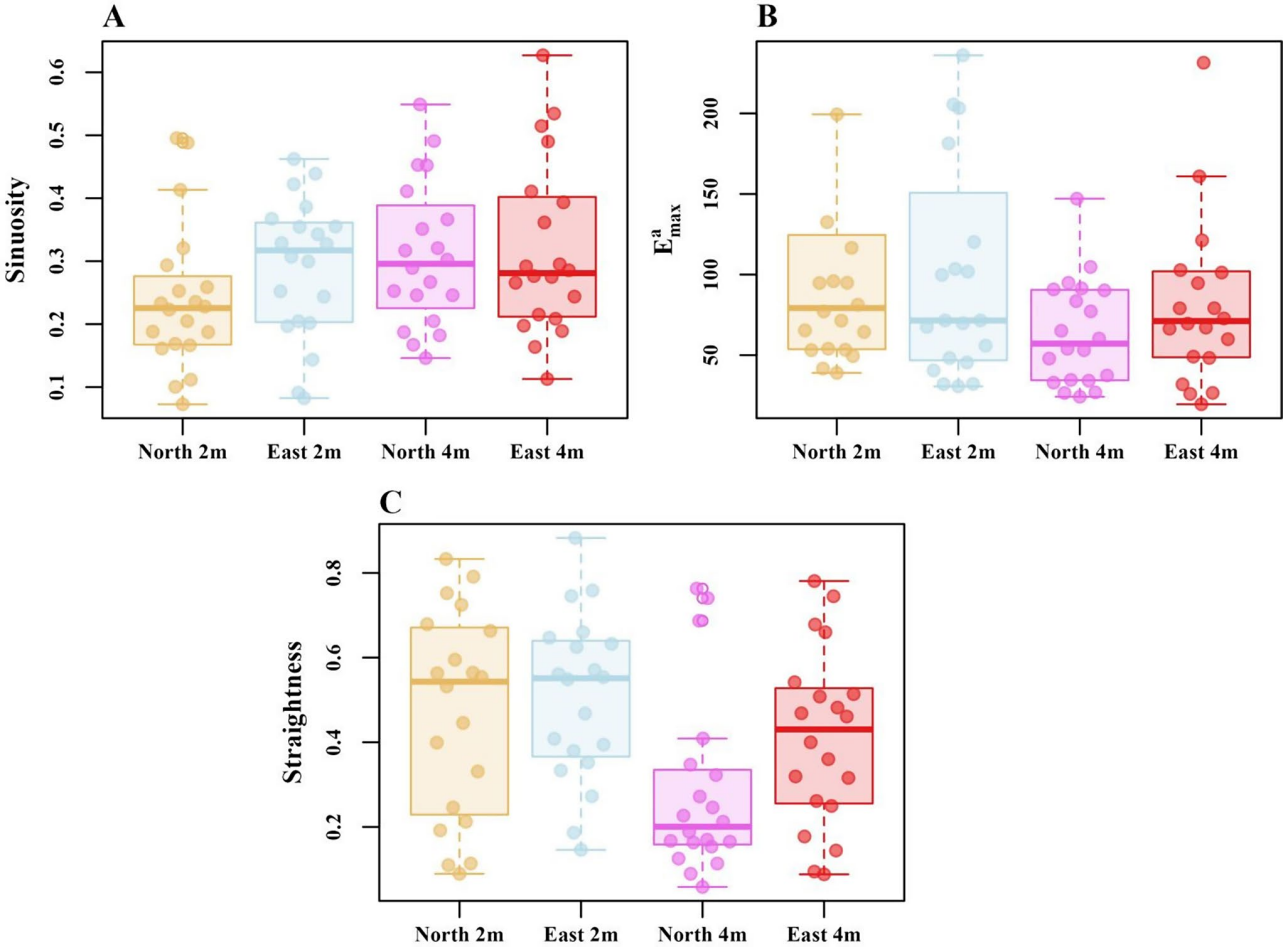
Path characteristics of ants at different displacement locations: (**A**) *sinuosity*, (**B**) ${E}_{max}^{a}$ and (C) *straightness*. Box plots display the median (line inside the box), interquartile range (box), and extreme values excluding outliers (whiskers). Individual data points are shown as dots

**Fig. 7:**
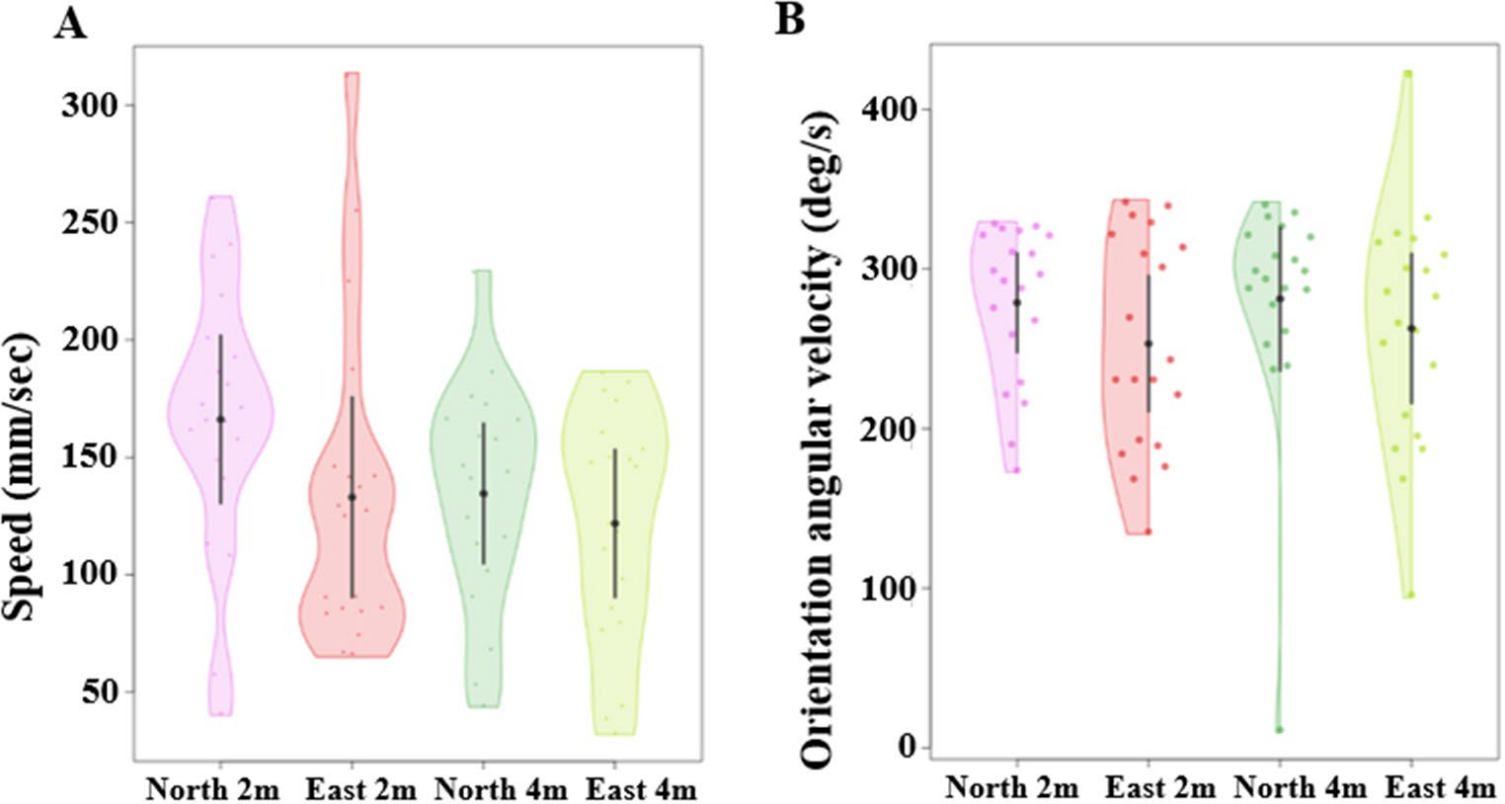
Comparison of speed and orientation angular velocity of ants at different displacement locations. The violin plots show the mean speed of ants across their entire trajectory at the displacement locations (**A**). The half-violin plots show the distribution of bootstrapped differences of mean orientation angular velocity of ants at the various displacement locations (**B**). In (**A**) and (**B**), the solid dot shows mean, while the vertical bar shows 95% confidence interval of the mean

We checked whether ants showed any differences in their path characteristics from oriented and non-oriented displacement locations. Firstly, in tortuosity ants appear to show lower *sinuosity* in the 4mN tests compared with the other conditions. However, the generalized linear model ANOVA showed no significant differences in *sinuosity* across the four displacement conditions (Z_(3, 76)_ = 0.64, *p =* 0.84), (Fig. 6A). Secondly, ${\text{E}}_{\text{m}\text{a}\text{x}}^{\text{a}}$ appeared similar across conditions. The generalized linear model ANOVA showed no significant difference in ${\text{E}}_{\text{m}\text{a}\text{x}}^{\text{a}}$ across the four displacement conditions (Z_(3, 76)_ = 1.05, *p* = 0.061), (Fig. 6B). Finally, with straightness, the 4mN displacement condition appeared to be lower on average than the other conditions (Fig. 6C). The generalized linear model ANOVA showed a significant difference in *straightness* across the four conditions (Z_(3, 76)_ = 12.59, *p* = 0.0001), but the Tukey post-hoc comparisons, however, showed no significant differences between any pair of displacement conditions (Table 2). On the whole then, path characteristics at different locations were similar, or differences were at most idiosyncratic, as we did not find any pairwise significant contrasts in any variable. During the displacement test, we did not find any noticeable impact of test location on the speed and angular velocity of the ants (Fig. 7A and B). The generalized linear-model ANOVA showed no significant difference between the displacement locations in mean speed (Z _(3, 76)_ = − 0.52, *p* = 0.61) and in the angular velocity of orientation-direction change (Z _(3, 76)_ = 0.8, *p* = 0.98).

**Table 2:** Tukey post hoc comparisons of statistical results for straightness between the displacement locations (alpha = 0.01)

Straightness				
Condition	ratio	SE	Z ratio	*p* value
east2m / east4m	1.228	0.576	0.437	0.9721
east2m / north2m	1.078	0.489	0.167	0.9984
east2m / north4m	1.802	0.948	3.303	0.0078
east4m / north2m	0.878	0.419	-0.272	0.993
east4m / north4m	1.468	0.803	0.702	0.8965
north2m / north4m	1.671	0.891	2.764	0.0308

**Fig. 8:**
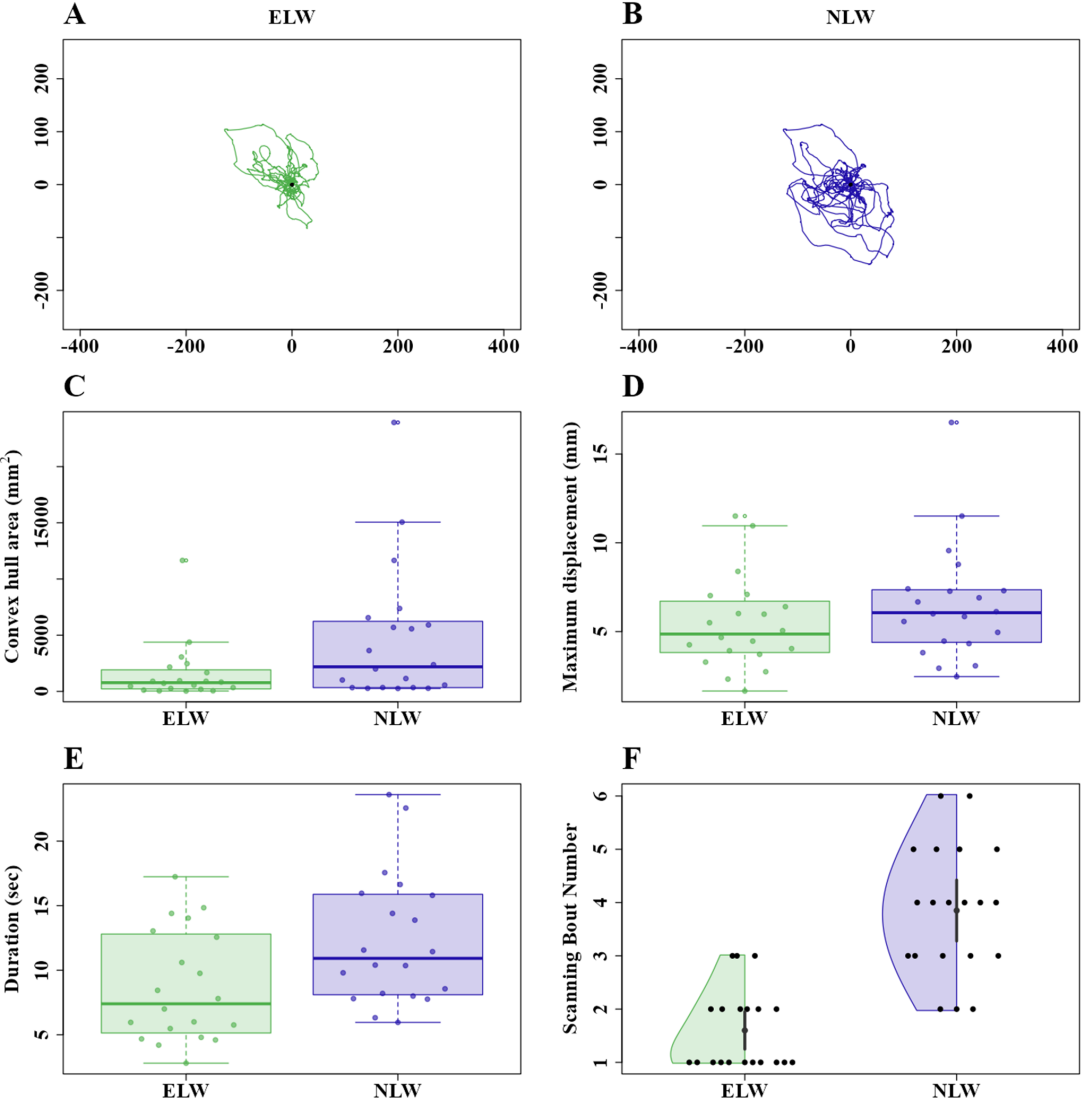
Characteristics of first learning walks of naive excavators and naive foraging ants at the nest. The line graphs show the first learning walks of excavators (**A**) and foragers (**B**). **C**) Convex hull area, **D**) maximum displacement from the nest, **E**) duration of the walk and **F**) Number of scans during the learning walks. The boxes indicate the median and quartiles, while the whiskers show extreme values excluding outliers. Each point represents a single trajectory. ELW denotes the excavators’ learning walk whereas NLW represents the first learning walk of foragers

We compared the naive learning walks of excavators with first learning walk of foragers (Fig. 8A and B). The excavators’ walk exhibited a smaller area compared to the naive foragers’ walks (Fig. 8C). But the t-test on convex-hull area failed to reach significance (t = − 2.09, df = 25.81, *p* = 0.045). In maximum displacement from the nest, the learning walks of naive foragers showed numerically larger values than did the excavators’ learning walks (Fig. 8D). However, the t-test revealed no significant difference (t = − 1.20, df = 36.01, *p* = 0.23). During the first learning walks, naive foragers stayed for a longer duration outside the nest than did the excavators (Fig. 8E). But the t-test again just failed to reach significance level (t = − 2.43, df = 36.95, *p* = 0.019). In learning walks, future excavators performed a smaller number of scanning bouts compared to future foragers. The t-test in this case revealed a significant difference (t = 4.43, df = 27.9, *p* = 0.001).

## Discussion

In summary, our observations of nest excavators’ behaviours in *Melophorus bagoti* showed that before becoming a nest excavator, workers made a single learning walk around the nest. This first walk of naive excavating ants was close to the nest, short in duration, and covered only a small area, similar to the first learning walk of foragers. On the next trips, ants started excavating the nest, carrying excavated sand in their mandibles within 5–10 cm around the nest and tossing the grain in a stereotypical way before returning straight to the nest. This activity lasted a shorter time and covered a smaller area than did their learning walks, and the ants moved faster and oscillated their gaze directions faster than they did on their learning walks. Other work that we are still analysing suggests that gaze swings are common when ants navigate homebound or outbound. The faster orientation swings on excavation walks may be coupled with the faster speed of walking. After the first excavation trip, when ants were displaced 2 m and 4 m North and East from their nest, they could orient towards the nest direction from 2 m North and East but not from 4 m away, suggesting that a catchment area between 2 and 4 m had been learned in one walk in our setting, similar to results after the first, naive learning walks of foragers. These latter ants could also orient nestward from 2 m but not from 4 m (Deeti and Cheng 2021b). Despite these differences in orientational performance, path characteristics at all the test sites were on the whole similar. As discussed further below, this similarity perhaps reflects the similarity across all the locations in their rotational image difference functions when compared to the panoramic image at the nest. In summary, ants perform a learning walk before they start excavating the nest and they generalize the views from that single learning trip to locations 2 m away from the nest.

The phenomenon of learning walks and learning flights as a precursor to the transition to foraging is well established across various hymenopteran species, including honeybees (Becker 1958; Capaldi and Dyer 1999; Capaldi et al. 2000; Lehrer 1991, 1993; Vollbehr 1975), bumblebees (Hempel de Ibarra et al. 2009), wasps (Stürzl et al. 2016; Zeil 1993a, b), wood ants (Judd and Collett 1998; Nicholson et al. 1999), and desert ants (Deeti and Cheng 2021a; Fleischmann et al. 2016; Müller and Wehner 2010). Through multiple flights or walks around their nest, these insects engage in exploratory behaviour, progressively covering more distance and exploring a wider range of directions to enhance their spatial knowledge around the nest (Capaldi et al. 2000; Deeti and Cheng 2021b; Fleischmann et al. 2016; Freas et al. 2019; Zeil and Fleischmann 2019). It is noteworthy that this exploration often includes walks or flights in all quadrants around the nest over successive trips. We have discovered that another outdoor performer, the excavator, also engages in a single learning walk around the nest area before undertaking their work. This seems surprising because excavating ants deposit excavated sand within a 5–10 cm area around the nest. It is likely that this learning walk still serves to facilitate the return trip on such a short journey. Possible functions include calibrating the sky compass and odometer (Wehner 2020) for path integration and also learning the visual scene surrounding the nest. Using a combination of path integration and view-based homing might expedite the journey home. Testing the function of the learning walk is not easy because our observations suggest that the workers will not excavate without first taking a learning walk. At the moment, we have no way to induce them to take an excavating trip without any learning walks.

The naive learning walk of red honey ants engaged in excavation activities displays parallels with the first learning walks of desert ant (*Cataglyphis*, *Melophorus*) foragers (Fleischmann et al. 2016; Deeti and Cheng 2021b). Comparing *Melophorus bagoti* would-be excavators and would-be foragers, the durations, lasting less than a minute, maximum displacement (less than 30 cm) and area covered (~ 20 cm^2^) are similar, or at least not significantly different. Would-be foragers, however, perform more than one learning walk before setting off to forage. Given that foraging takes place at a much greater distance than excavating, the multiple learning walks make functional sense. Multiple walks are needed to return from long distances. After all, a single learning walk is good for returning only from ~ 2 m away (Deeti and Cheng 2021b). However, multiple learning walks up to 1 m distance in all directions from the nest entrance allowed them to orient nestwards from 10 m distance (*Cataglyphis*: Fleischmann et al. 2018; *Melophorus*: Wystrach et al. 2012; Deeti et al. 2020). In essence, the multiple learning walks serve to increase the catchment range for the ants. By exploring and learning the surroundings in various directions from the nest entrance, the ants expand their spatial knowledge, allowing them to navigate and return from more extended distances with precision. This adaptive strategy ensures efficient orientation and successful homeward journeys, especially in the context of foraging activities that necessitate exploration over more significant distances.

Unfamiliarity is thought to trigger ‘uncertainty’ in solitary foraging ants, although how and what is encoded about uncertainty remains unclear. Experimental manipulations have included changes in the visual panorama and conflicts between path integration and view-based navigation. These alterations manifested as a reduction in the path straightness of foragers, indicating one response to navigational uncertainty induced by visual changes (Wystrach et al. 2019, 2020; Islam et al. 2021; Deeti et al. 2023b). Another change is slower travel speeds (Buehlmann et al. 2018). In the current study, path characteristics were on the whole similar across displacement-test locations, even though the ants were homeward oriented in their initial trajectories at 2 m distance but not at 4 m distance. Our image analysis of panoramic views at the test sites perhaps provides an explanation. When compared with the panoramic view at the nest, the views at all test sites differed from the nest view by similar amounts on a pixel-by-pixel basis. A pixel-by-pixel analysis is unlikely to the be sole analysis carried out by the ants’ visual system, which is known to abstract features such as the skyline where terrestrial objects meet the sky (Graham and Cheng 2009) and the fractional position of mass, the proportion of the visual scene to the left and right of the goal direction (Lent et al. 2013). Nevertheless, the pixel-by-pixel analyses serve to give proxy measures of similarity to the panoramic view at the nest.

What the image analysis does not explain is why ants were homeward oriented in initial trajectories at 2 m but not at 4 m, when all the panoramic views contained a minimum-mismatch best direction pointing roughly at the nest direction. By the image analysis, the ants should have been able to orient homewards from 4 m as well as at 2 m. It is possible that other modalities of cues are used as well, such as olfactory (Buehlmann et al. 2015), vibratory (Buehlmann et al. 2012), or magnetic cues (Buehlmann et al. 2012), and these cues are less helpful at 4 m compared with 2 m, but we have no evidence for the use of these cues in this study or for this species. Although we think it unlikely, it is possible that ants can home from 2-m distance from the nest even without any learning experiences at all. It remains unexplained why our ants could not orient homewards from 4 m.

In conclusion, *M. bagoti* ants engage in learning walks in the proximity of the nest before taking on the role of a nest excavator. These learning walks extend up to 15 cm away, allowing the ants to acquire spatial knowledge about their surrounding panorama up to at least 2 m away from the nest. Future studies should delve into a more detailed exploration of the differences and similarities in the behaviour of different worker castes of naive ants, based on their specific role in the colony, such as guarding the nest, foraging, dumping and excavating activities. Additionally, there is a need for an examination of the underlying neurological processes that support learning walks in ants. This research avenue holds the potential to enhance our understanding of the intricate mechanisms governing ant behaviour and learning strategies.

## Electronic supplementary material

Below is the link to the electronic supplementary material.

Supplementary Material 1

## Data Availability

Supplementary videos, Excel file and R scripts are available at Open Science framework: https://osf.io/gqb8r/.
