# Supplementary material for: Nest excavators’ learning walks in the Australian desert ant Melophorus bagoti
Source: Anim Cogn. 2024 May 24;27(1):39. doi: 10.1007/s10071-024-01877-3 (PMC11126504; doi:10.1007/s10071-024-01877-3)
Supplement: Supplementary file 1 — Supplementary Material 1 [file 10071_2024_1877_MOESM1_ESM.docx]

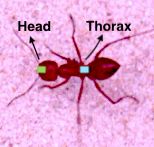


**Figure S1.** The annotated ants’ two body positions, used in data analysis: Front of the head and middle of the thorax.
